# Correction: Upregulation of DNA repair genes and cell extrusion underpin the remarkable radiation resistance of *Trichoplax adhaerens*

**DOI:** 10.1371/journal.pbio.3003132

**Published:** 2025-04-08

**Authors:** Angelo Fortunato, Alexis Fleming, Athena Aktipis, Carlo C. Maley

The last two authors, Athena Aktipis and Carlo C. Maley, should be noted as contributing equally to this work.
