# Supplementary figures and images for: Upregulation of DNA repair genes and cell extrusion underpin the remarkable radiation resistance of Trichoplax adhaerens
Source: PLoS Biol. 2021 Nov 17;19(11):e3001471. doi: 10.1371/journal.pbio.3001471 (PMC8635375; doi:10.1371/journal.pbio.3001471)

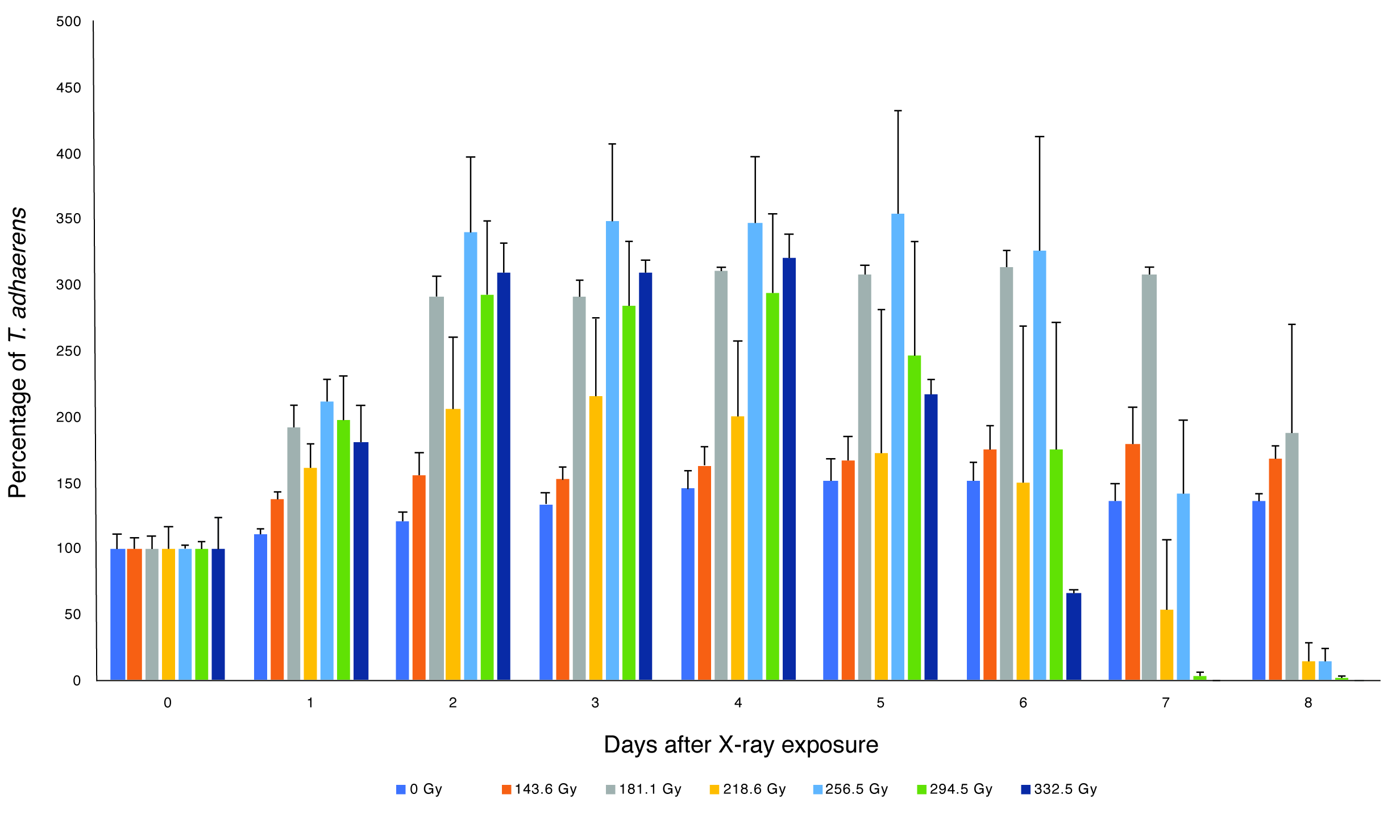

Supplement: S1 Fig — All radiation doses induce an increased number of T. adhaerens. There is a statistically significant positive correlation between the doses of radiation and the number of T. adhaerens calculated as the average of the first 4 days before the beginning of animal death caused by radiation (Pearson correlation, r = 0.814, P = 0.026). All the doses with the exception of 143.6 Gy determine a sharp decrease in the number of animals 8 days after the X-ray exposure. The 8 days final time point is because after 8 days, the environmental plates’ condition deteriorates (for instance, reduction of algae and weather quality), and the transferring of animals in fresh plates could compromise their integrity, in particular of the radiation treated ones. Histograms represent the mean ± SEM (error bars). The data used to generate this figure can be found in S1 Data. (TIF) [file pbio.3001471.s001.tif]

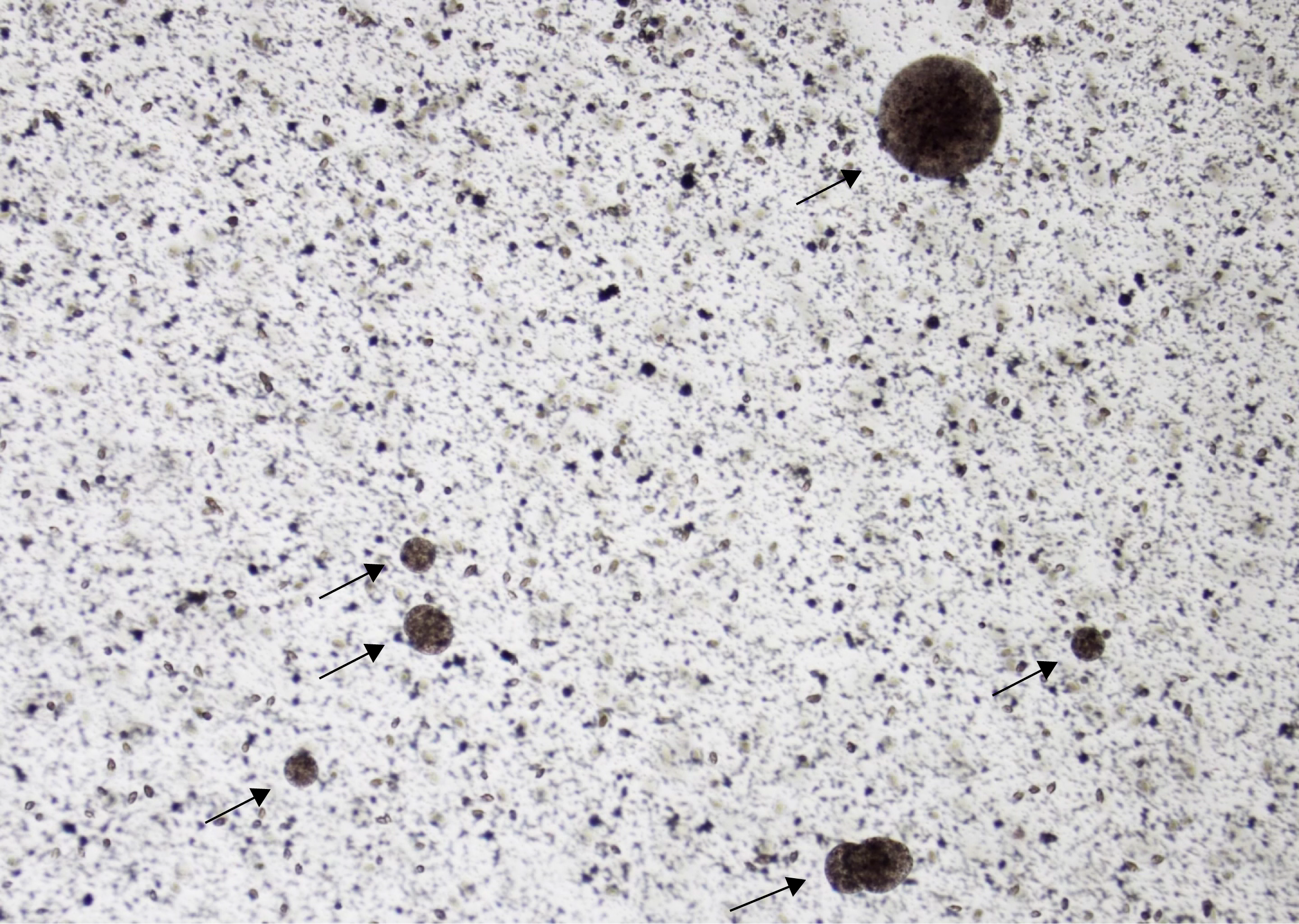

Supplement: S2 Fig — (TIF) [file pbio.3001471.s002.tif]

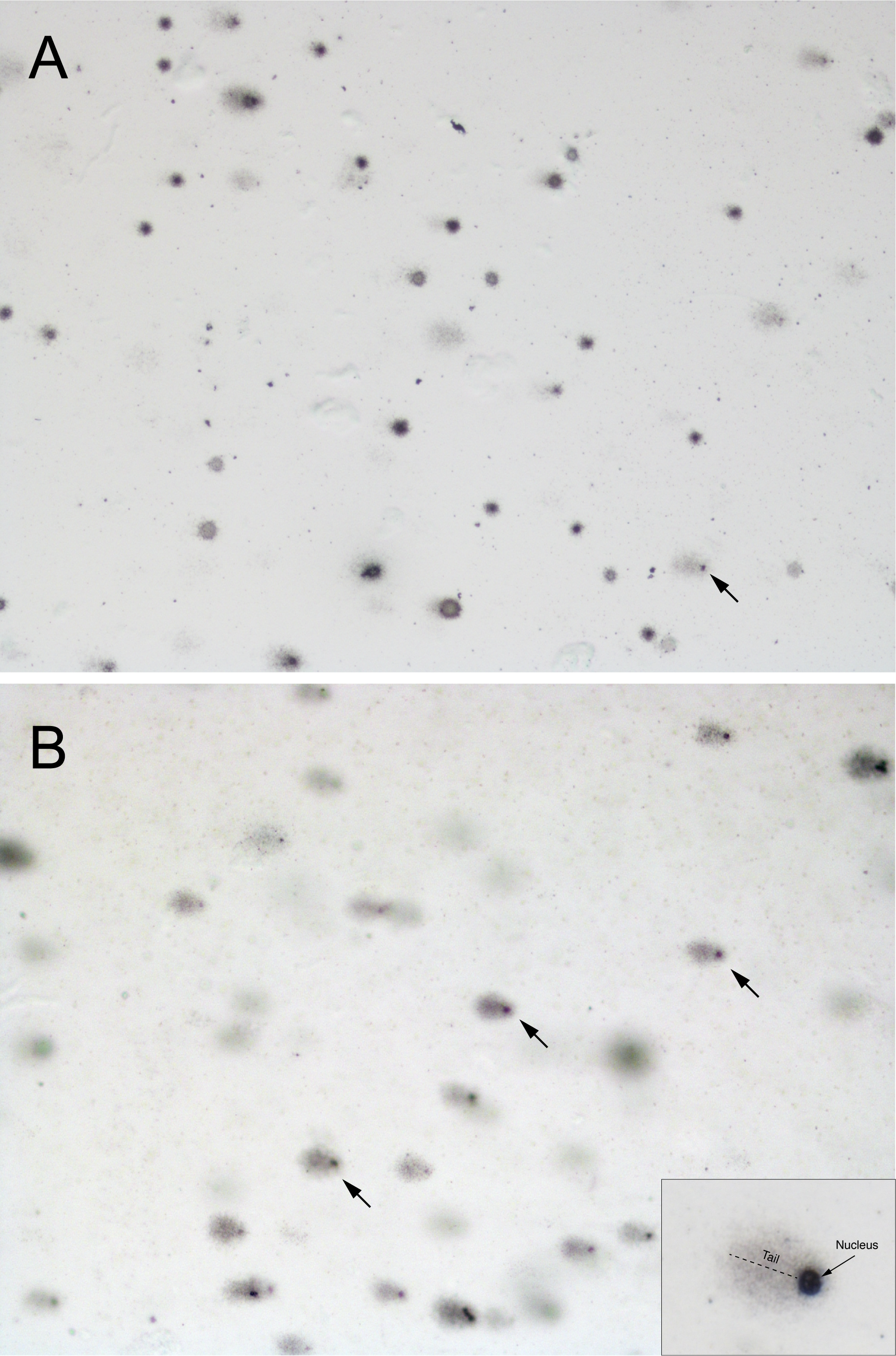

Supplement: S3 Fig — (A) The controls have few nuclei showing DNA fragmentation. (B) In contrast, animals exposed to 143.6 Gy of X-rays have many more nuclei with extensive fragmentation of their DNA (Mann–Whitney U Test, P < 0.0001). The arrows indicate examples of a “comet” with the nucleus containing unfragmented DNA and the electrophoretic migration of fragmented DNA (tail, shown in the inset of panel B). (TIF) [file pbio.3001471.s003.tif]

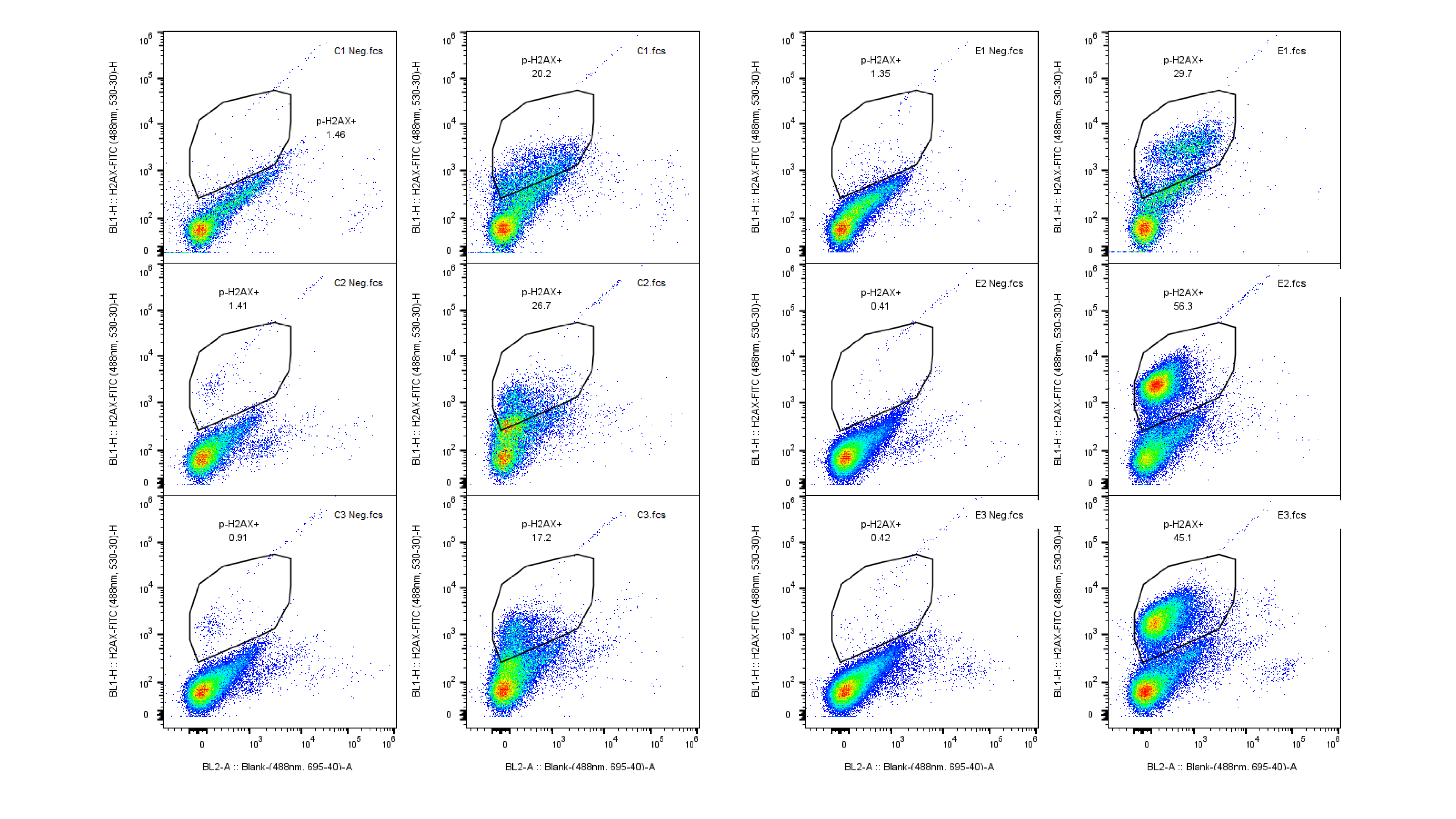

Supplement: S4 Fig — We confirmed DNA damage using the H2AX phosphorylation assay, controls (A, B), experimental (C, D) cells. The solid line shows the region of cell-derived fluorescence signals. The data used to generate this figure can be found in S4 Data. (TIF) [file pbio.3001471.s004.tif]

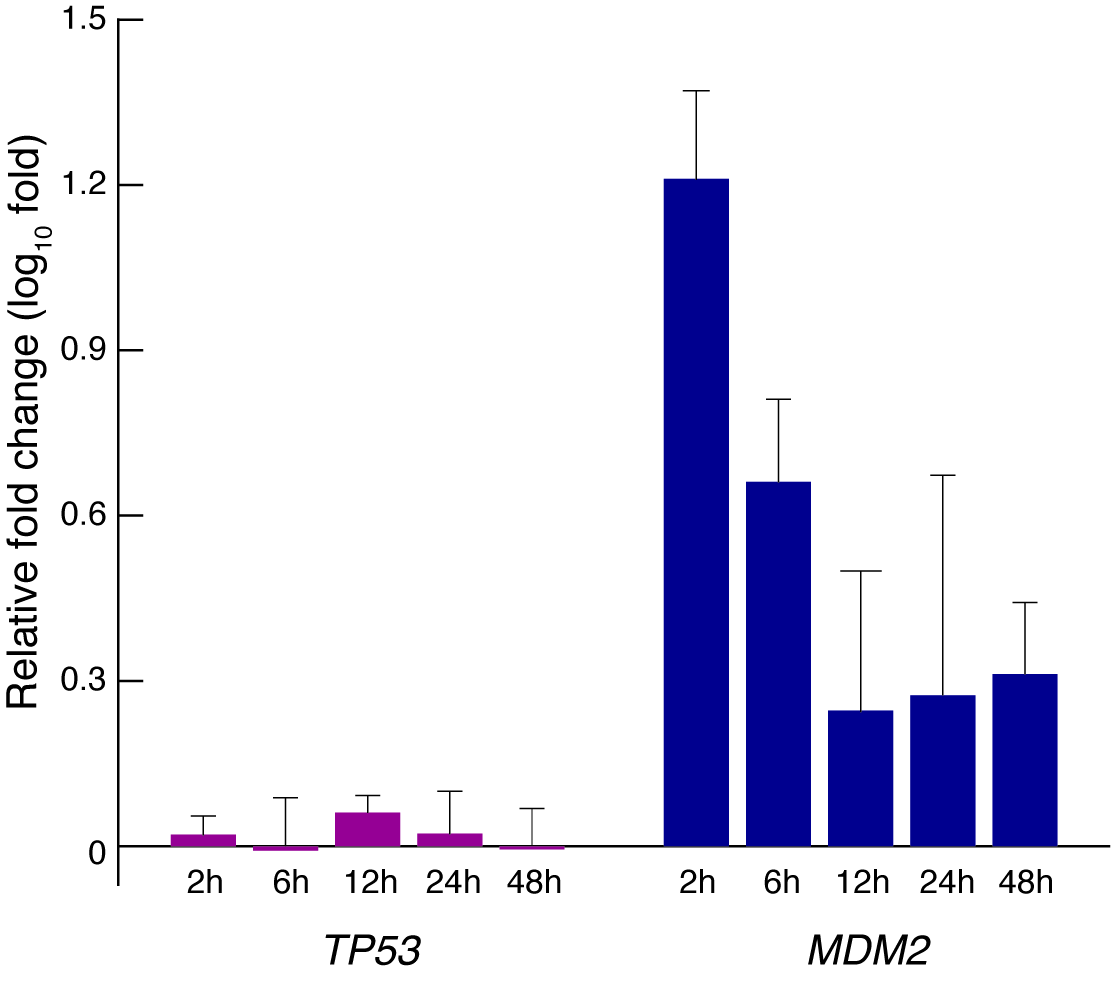

Supplement: S5 Fig — Each experiment was repeated thrice (Mann–Whitney test, MDM2 vs control, P < 0.05; TP53 vs control, P = NS; TP53 vs MDM2, paired t test, P < 0.05). Histograms represent the mean (log10 fold) ± SEM (error bars). The data used to generate this figure can be found in S5 Data. (TIF) [file pbio.3001471.s005.tif]
